# The diagnosis and treatment in patients with a bipolar fracture–dislocation of the forearm: a retrospective study

**DOI:** 10.1186/s13018-022-03278-z

**Published:** 2022-08-12

**Authors:** Maoqi Gong, Hanzhou Wang, Xieyuan Jiang, Yang Liu, Junlin Zhou

**Affiliations:** 1 Department of Orthopedic Surgery, Beijing Jishuitan Hospital, Beijing, 100035 People’s Republic of China; 2 Department of Orthopedic Surgery, Beijing Chaoyang Hospital, Capital Medical University, 8 Gongren Tiyuchang Nanlu, Chaoyang District, Beijing, 100020 People’s Republic of China

**Keywords:** Forearm injuries, Elbow dislocations, Fractures, Diagnosis, High-energy injury

## Abstract

**Backgrounds:**

This study aims to investigate the treatment and clinical effect of bipolar fracture–dislocation of the forearm.

**Methods:**

From March 2011 to September 2021, patients with bipolar fracture–dislocation of the forearm admitted to XXX and XXX Hospital were retrospectively analyzed. The timing of rehabilitation depended on the joint stability after the operation. The forearm function was evaluated according to the Anderson forearm function score.

**Results:**

A total of 40 patients who underwent surgical treatment were screened, but only 24 received a minimum of 6 months of follow-ups and were included in the study. Nineteen males and five females were enrolled in the study, with an age range of 18–65 years and an average of 40.4 years. With an average follow-up of 23.6 months (7–62 months), no case was related to functional malformations and infections. The average range of motion of flexion and extension at the elbow was 125.9° (98°–138°), the average range of motion of flexion and extension at the wrist was 144.2° (120°–156°), and the average range of motion of rotation at the forearm was 139.6° (88°–170°). The Anderson's forearm function score of the last follow-up presented: excellent in 16 cases, satisfactory in 6 cases, dissatisfactory in 1 and failure in 1.

**Conclusions:**

Bipolar fracture–dislocation of the forearm always represents high-energy injuries, of which the treatment principle includes complete reduction in distal and proximal dislocations and rehabilitation training as early as possible. Intraoperative fracture fixation follows after a stable reduction in the dislocation.

## Introduction

Bipolar forearm fractures are considered a rare and severe traumatic pattern consisting of ulna and radius fractures [1]. Forearm fractures are always accompanied by dislocation of the elbow and wrist due to tearing of the soft tissue, especially the interosseous membranes. With prolonged immobilization and inappropriate choices of internal fixation, the affected forearm fails to achieve an excellent clinical outcome, especially with severe rotation limitations influencing activities of daily living [2].

Odnea et al. first proposed the concept of bipolar fracture–dislocation of the forearm in 1952, in which radiographs showed that Monteggia fracture and Galeazzi fracture occurred in the same forearm [3–5]. However, cases matching these criteria were infrequent [6]. In 1994, Jupiter reported 10 patients with a complex forearm fracture–dislocation but without a concomitant Monteggia fracture and Galeazzi fracture [7]. Subsequent analysis of the common characteristics of fracture led to the term “floating radius,” where proximal and distal ulnar-radial joint dislocation/subluxation and various types of forearm fractures occur concurrently. In addition, he emphasized that soft tissue between the radius and ulna played an essential role in preventing the radius from “floating.”

This study further proposed that the forearm should be maintained in a supination position for 6 weeks postoperatively to prevent the interosseous membrane from contracture [7]. However, these patients had diverse clinical outcomes because of the unstandardized management of fracture–dislocation. Subsequent articles on the bipolar injury of the forearm were rare and of all case reports [8–10]. Those work focused on the elbow and wrist dislocation or fracture–dislocation, part of the “floating forearm” rather than the “floating radius.” Therefore, we designed a retrospective study to assess the treatments and clinical outcomes in patients with a bipolar fracture–dislocation of the forearm.

## Methods

### Participants

From March 2011 to September 2021, 40 patients diagnosed as bipolar fracture–dislocation of the forearm were retrospectively collected. The Ethics Committee of the XXX institution has approved this study, and all patients have confirmed and signed informed consent.

The inclusion criteria were as follows: (i) patients aged ≥ 18 years with proximal and distal radioulnar joints dislocation/subluxation and with one or more fractures of the ulnar and radius; (ii) patients with a complete postoperative follow-up of more than 6 months. Exclusion criteria: (i) patients with conservative treatments; (ii) patients diagnosed as classic Essex-Lopresti injury: a particular type of bipolar forearm injury that has been widely recognized [11]; (iii) patients diagnosed as Criss-Cross injury: a specific type of bipolar injury to the forearm [12]; (iv) patients with “floating forearm” injury, a type of injury with both elbow and wrist joint dislocation or fracture–dislocation; and (v)patients with deformity, severe dysfunction and infectious diseases.

Baseline demographic data and trauma history are collected and presented in Table 1.

**Table 1:** Characteristics of patients undergoing bipolar fracture–dislocation of forearm

No.	Gender	Age	Side	Causes of injury	Injury type	Treatment	Excursus
1	Male	38	Left	Fall	A	ORIF middle ulna; distal radius	
						Plaster external fixation distal radius	
2	Male	28	Right	Machine accident	A	ORIF ulna: olecranon, shaft; distal radius	Fracture supracondylar humerus
3	Male	48	Left	Fall	A	ORIF middle ulna; distal radius	
						Plaster external fixation distal radius	
4	Female	65	Right	Fall	A	ORIF middle ulna; distal radius	
5	Male	38	Right	Fall	A	ORIF ulna: shaft, olecranon; distal radius	
						Replacement of radial head	
6	Male	45	Left	Traffic Accident	A	ORIF middle ulna; distal radius	
7	Male	45	Left	Fall	A	ORIF middle ulna; distal radius	
						Screws ulna: coronoid process	
8	Male	42	Right	Traffic Accident	B	ORIF middle ulna	
						K wire DRUJ	
						Repair lateral ligament of elbow	
						Hinged external fixation DRUJ	
9	Male	41	Right	Fall	B	ORIF middle ulna; distal radius	
						K wire PRUJ	
10	Male	42	Right	Fall	B	ORIF middle ulna; distal radius; radial head	Postoperative complications with heterotopic ossification and elbow stiffness
						Release and external fixation of elbow (secondary treatment)	
11	Male	45	Right	Machine accident	B	ORIF middle and distal ulna	Open fracture
						ORIF middle and distal radius	
						Debridement of elbow	
12	Male	44	Left	Fall	B	ORIF ulna: olecranon	Fracture proximal humerus
						ORIF distal radius; radial head	
13	Female	46	Right	Fall	C	ORIF middle ulna	
						K wire PRUJ and DRUJ	
14	Male	25	Right	Fall	D	ORIF middle radius	
						Hinged external fixation elbow	
15	Male	42	Right	Fall	D	ORIF middle radius	
						Plaster external fixation distal radius	
16	Male	25	Left	Fall	D	ORIF middle radius	
						K wire DRUJ	
17	Male	18	Right	Fall	E	Traction reduction elbow	
						Plaster external fixation elbow and wrist	
18	Male	28	Left	Fall	E	ORIF distal ulna; distal radius	
						Hinged external fixation elbow	
						Repair medial and lateral ligament of elbow	
19	Female	40	Left	Fall	E	ORIF distal ulna; distal radius	
						Replacement of radial head	
						Repair lateral ligament of elbow	
20	Male	40	Left	Fall	E	ORIF distal radius	Fracture scaphoid and lunate
						K wire ulna: coronoid process	
21	Male	47	Left	Traffic Accident	E	ORIF ulna: coronoid process; distal radius	
						Repair lateral ligament of elbow	
22	Female	53	Left	Fall	E	K wire ulna: distal radius	Open fracture
						Hinged external fixation wrist	
						Repair lateral ligament of elbow	
						Debridement of radius	
23	Male	50	Left	Fall	F	ORIF distal radius	
						Hinged external fixation wrist	
						Repair lateral ligament of elbow	
24	Female	35	Left	Fall	F	ORIF distal radius	
						Replacement of radial head	
						Repair lateral ligament of elbow	
						Plaster external fixation elbow	

*ORIF* open reduction and internal fixation; *K wire* Kirschner wire; *PRUJ* proximal radioulnar joint; *DRUJ* distal radioulnar joint

### Preoperative evaluation

Besides the routine medical treatments, patients with high-energy trauma were examined immediately for swelling, tension, nerve and blood supply to exclude osteofascial compartment syndromes. Surgical debridement with intravenous antibiotics was performed in the emergency room for patients with an open fracture.

### Treatment principles

The treatment of proximal and distal fracture–dislocation was as follows: (i) Divergent elbow dislocations were reduced by traction under anesthesia, which often achieved an excellent reduction outcome [13–16]; convergent elbow dislocation always caused instability of the radial head. Therefore, open reduction through a lateral elbow approach was required to repair the annular ligament [17–22]. (ii) Ulnar shaft fractures were treated with open reduction and internal fixation along the axis of the ulna; Shaft and distal radius fractures surgery were performed through the Henry incision; Kirschner wires or external fixators were also alternative options. (iii) For Monteggia fracture Bado I, it was necessary to repair the annular ligament when the radial head was still unstable [23, 24]; similarly, for Monteggia fracture Bado II, double-check for the stability of the lateral ulnar collateral ligament was required [25–27]. (iv) Radial head dislocation in Monteggia fracture Bado II was associated with fractures and posterolateral rotational instability, which required a repair of the lateral ulnar collateral ligament. (v) Patients with unstable elbow joints should receive hinged external fixators. The Kirschner wire or screw fixation placed 2–3 cm from the joint was used in patients with distal ulnoradial instability to maintain the affected limb in a supination position. Due to soft tissue insertion, reducing the proximal radioulnar joints was hard, which might receive an open reduction treatment [23, 24]. (vi) For the comminuted fracture of the radial head that could not be repaired anatomically, the radial head replacement was optional [28–31].

### Postoperative treatment and clinical evaluation

The starting time of rehabilitation depended on clinical outcomes. A stable humeroulnar joint without a hinged external fixator was avoided to motion within 2 weeks, and the extension range was gradually increased. The functional exercise started immediately for patients with satisfactory treatment of distal radioulnar joint and repaired lateral ulnar collateral ligament. For patients with anterior dislocation of the radial head, external fixation was not removed until achieving 90° elbow flexion, which maintained the affected limb in supination for 4 weeks. However, patients with instability of the radioulnar joint were treated with a cross-cutting pin for 4–6 weeks postoperatively. The hinged external fixator for the elbow was removed at 6 weeks postoperatively.

The range of motion, fracture union, and complications were evaluated postoperatively (Table 2). The Anderson’s forearm function score was used to assess the forearm function, including elbow and wrist joint activity at the last follow-up. This scoring system used four levels to evaluate the clinical outcomes [32]. Excellent: fracture healing, lost movement of flexion and extension in elbow and wrist joint < 10°, lost forearm rotation activity < 25%; satisfactory: fracture healing, lost movement of flexion and extension in elbow and wrist joint < 20°, lost forearm rotation activity < 50%; dissatisfaction: fracture healing, lost movement of flexion and extension in elbow and wrist joint > 30°, lost forearm rotation activity > 50%; and failure: malunion, nonunion, or uncured chronic osteomyelitis.

**Table 2:** Evaluation of clinical outcome (range of motion and Anderson’s score)

Number	Follow-up (months)	Range of motion			Anderson’s score
		Elbow (flexion and extension) (°)	Wrist (flexion and extension) (°)	Forearm (rotation)(°)	
1	9	138	156	170	Excellent
2	25	136	158	168	Excellent
3	13	136	152	168	Excellent
4	16	135	152	156	Excellent
5	29	125	153	135	Excellent
6	32	134	152	140	Excellent
7	35	135	150	150	Excellent
8	33	138	142	166	Excellent
9	32	133	136	150	Excellent
10	25	98	138	88	Failure
11	21	136	148	145	Excellent
12	62	135	150	146	Excellent
13	9	128	145	156	Excellent
14	12	110	120	113	Dissatisfactory
15	13	112	135	116	Satisfactory
16	7	135	150	135	Excellent
17	6	128	142	150	Excellent
18	15	118	140	112	Satisfactory
19	22	114	153	148	Excellent
20	28	114	135	126	Satisfactory
21	29	116	140	128	Satisfactory
22	22	117	132	120	Satisfactory
23	35	118	136	117	Satisfactory
24	37	132	145	147	Excellent

## Results

A total of 24 patients were enrolled in this study, with 19 males and 5 females, ranging from 18 to 65 years (the average age was 40.4 years). These patients’ left and right sides were injured in 13 and 11 cases, respectively. The causes of injury included falling from a great height (19 cases), traffic accidents (3 cases) and machine traumas (2 cases). Two cases were with open fractures.

By retrospectively summarizing the injury characteristics and surgical treatments of bipolar fracture–dislocation of the forearm, we divided up the enrolled patients into six conditions as follows:

Condition A anterior Monteggia fracture + Galeazzi fracture (or variant) (7 cases);

Condition B posterior Monteggia fracture + distal radius fracture + distal radioulnar joint dislocation (5 cases);

Condition C ulnar shaft fracture + dislocations of the proximal and distal radioulnar joint (1 case);

Condition D radial shaft fracture + dislocations of the proximal and distal radioulnar joint (3 cases);

Condition E divergent elbow dislocation + distal radius fracture + distal radioulnar joint dislocation (6 cases);

Condition F convergent elbow dislocation + distal radius fracture + distal radioulnar joint dislocation (2 cases).

All radius shaft fractures were treated with open reduction and internal fixation (ORIF). Closed reduction and plaster external fixation of distal radius fractures in 4 cases, single external fixation in 2 cases and Kirschner wires external fixation in 6 cases were reported; internal fixation of radial head fractures in 2 cases and the radial head replacement in 3 cases were included. Besides, ligament repair in 7 cases and hinged external fixators of the elbow in 3 cases were also recorded.

Patients were followed up for an average of 23.6 months (7–62 months). All cases treated with internal fixation showed great clinical outcomes without functional malformations and wound infection. The average activity of flexion and extension was 125.9° (98°–138°) for the elbow and 144.2° (120° ~ 156°) for the wrist. The range of motion of rotation was 139.6° (88°–170°) for the forearm. The Anderson’s forearm function score at the last follow-up showed: excellent in 16 cases, satisfactory in 6 cases, unsatisfactory in 1 case and failure in 1 case.

**Fig. 1:**
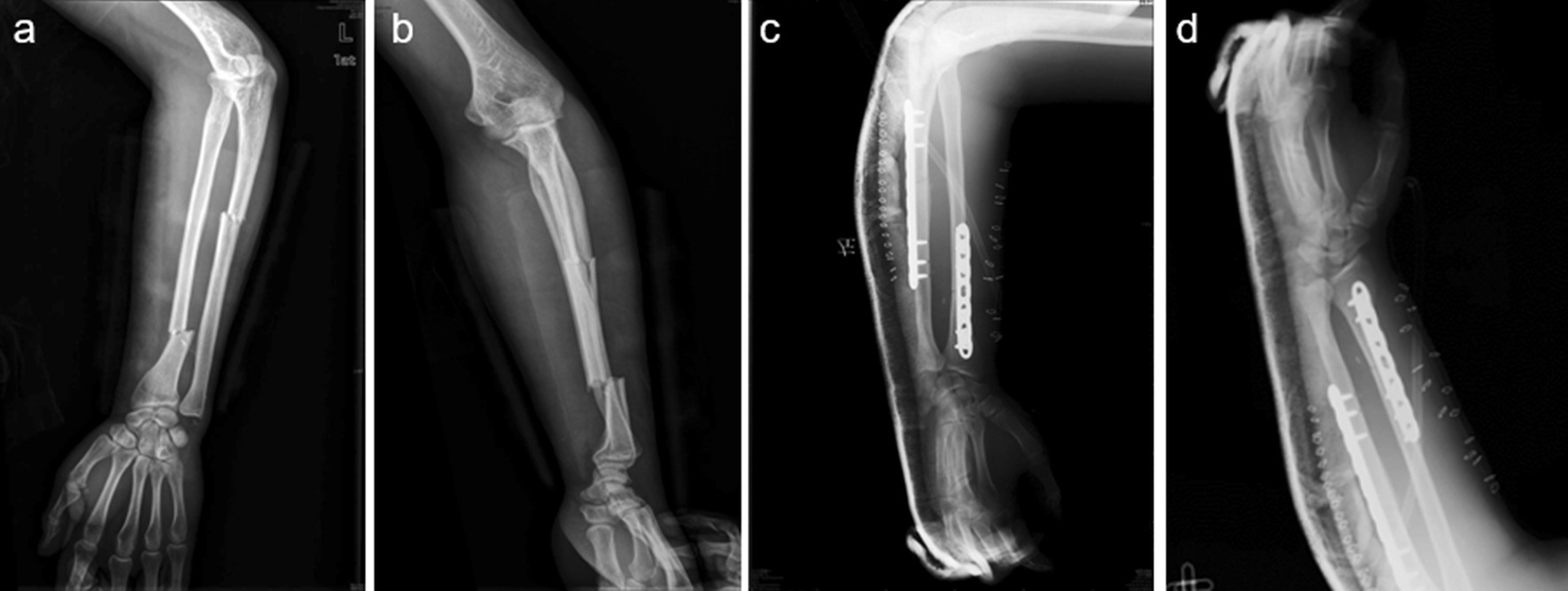
**a–b**: Preoperative radiographs show a classic Monteggia and Galeazzi fracture, also named “floating radius.” **c–d** This patient received an ORIF and plaster fixation with the forearm

**Fig. 2:**
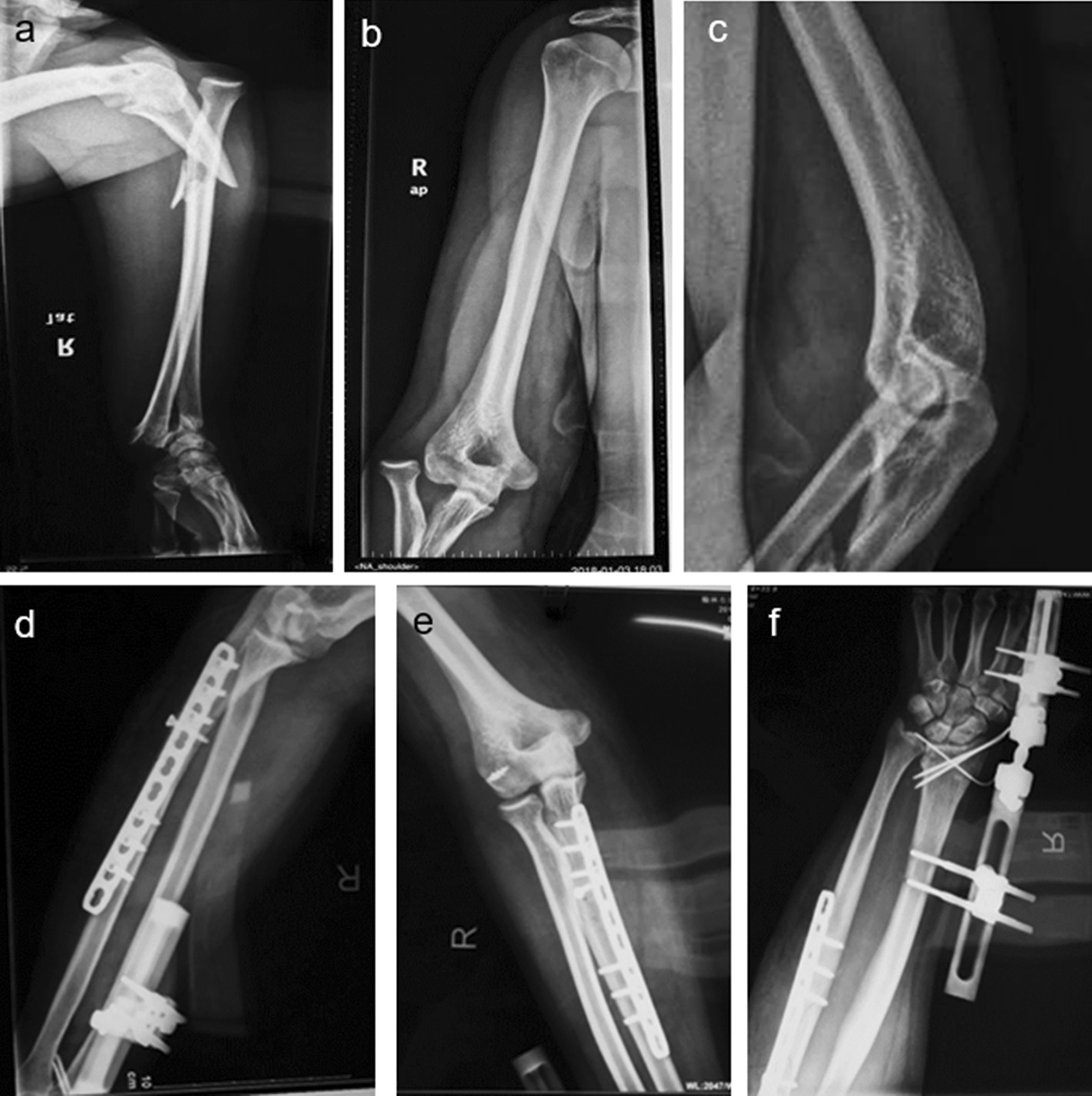
**a–c**: A 42-year-old right-hand dominant male case showed a Bado II C Monteggia fracture and distal radius fracture with dislocation of the distal radioulnar joint. **d–f**: The patient was treated with K wires and an external fixator, ORIF of the ulnar shaft and a repair of the lateral ligament of the elbow was also applied. Worth mentioning was that the external fixator was removed at 6 weeks postoperatively

**Fig. 3:**
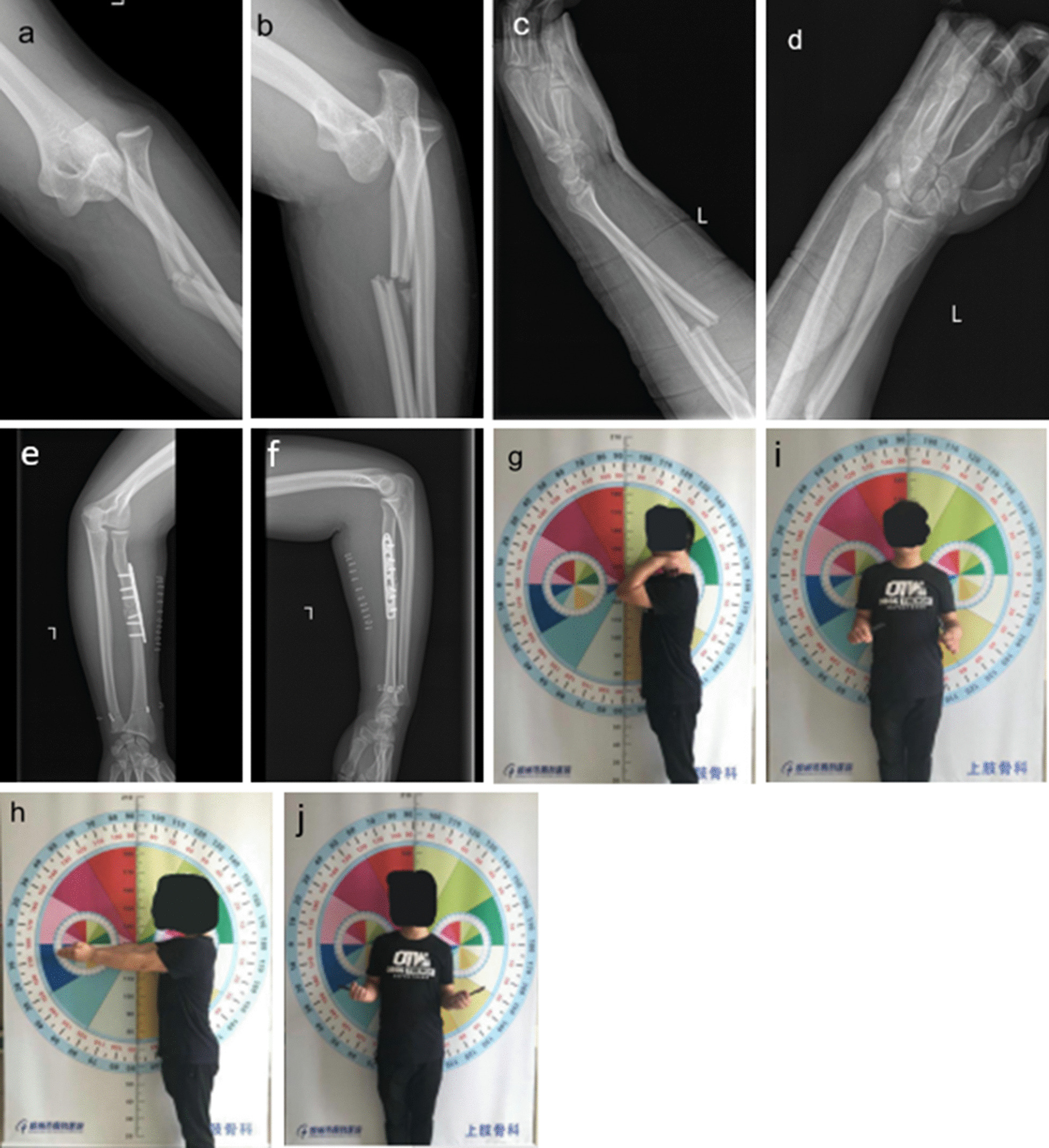
**a–d**: A 25-year-old male sustaining a radius shaft fracture combined divergent elbow dislocation and distal radioulnar joint dislocation. **e–f**: During operation, elbow dislocation was reduced by traction. Internal fixation of the radius shaft was performed using locking plates and reattached with an endobutton plate of the distal radioulnar joint. **g–j**: At the 7-month follow-up, the patient received an examination of the range of motion and an excellent clinical outcome compared with the opposite side

**Fig.4:**
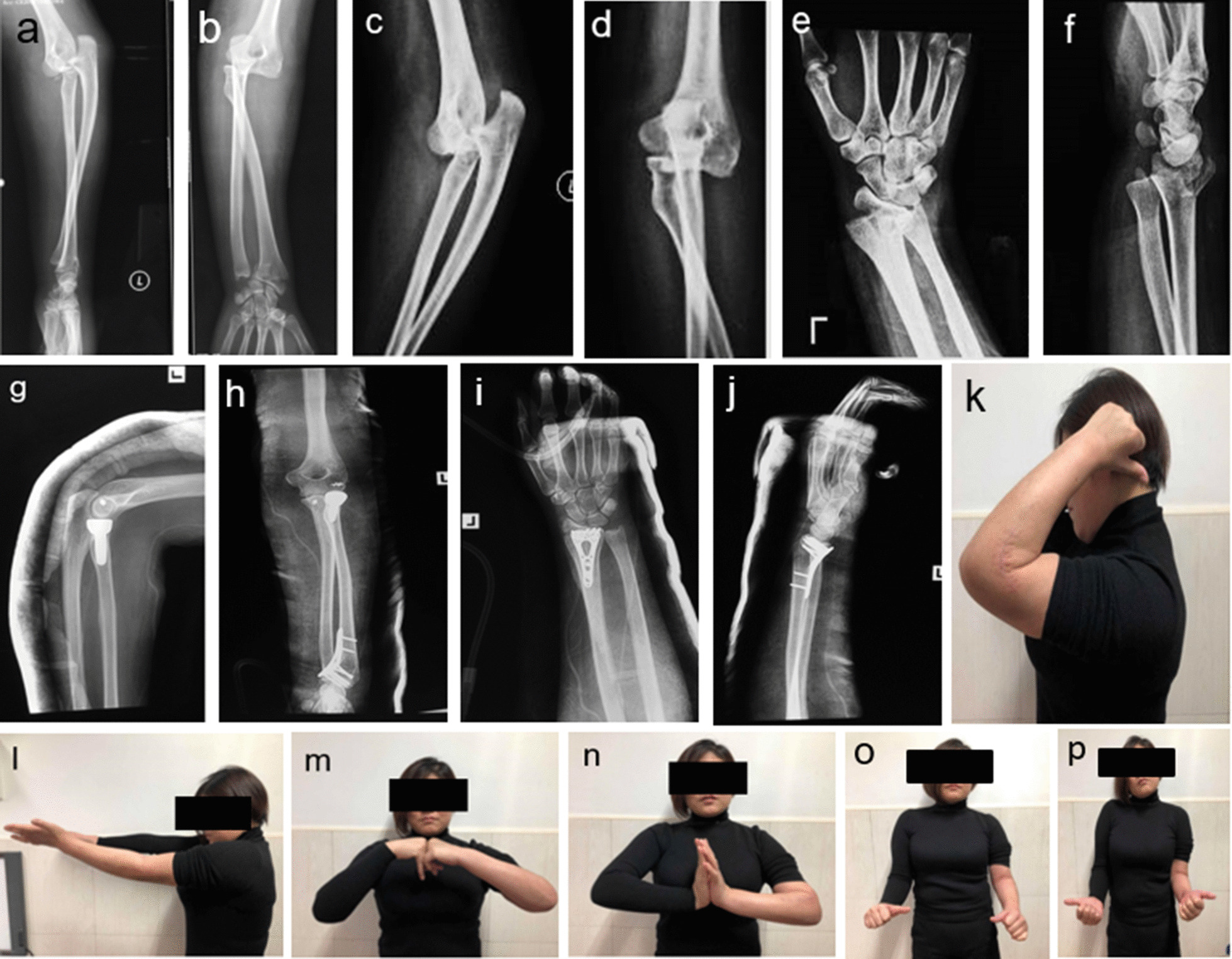
**a–f**: A 35-year-old female, whose preoperative radiographs demonstrated a distal radius and radius head fracture, fell from a two-story height. Furthermore, posterior dislocation of the proximal ulna with displaced radius head fracture was diagnosed as a sign of convergent elbow dislocation. **g–j**: With the failure of initial closed reduction under general anesthesia for elbow dislocation, open reduction was attempted to unlock the stuck radius head. ORIF of distal radius fracture followed after replacement of radius head and repair of lateral collateral ligament and joint capsule. **k–p**: At the latest follow-up after the injury, the patient had nearly full range of motion of her elbow and wrist

In order to introduce our surgical treatment, four cases are presented in Figs. 1, 2, 3 and 4.

Unusually, both Monteggia and Galeazzi fractures occurred on the same side in addition to an elbow dislocation, which was treated with a locking plate and a plaster fixation for the forearm (Fig. 1a–d).

A 42-year-old right-hand dominant male fell from a bicycle on his outstretched upper extremity with the elbow and wrist in extension (Fig. 2a–c). X-ray radiographs showed a Bado II C Monteggia fracture and distal radius fracture with dislocation of a distal radioulnar joint. It was hard to reduce the comminuted distal radius fracture immediately. Therefore, the patient was treated with Kirschner wires and an external fixator. ORIF of ulnar was performed by increasing backward radian slightly. In addition, repair of the elbow’s lateral ligament was also applicated (Fig. 2d–f). At 6 weeks postoperatively, the external fixator was removed and rehabilitation therapy was initiated.

A 25-year-old male who fell to his left side from a 5 m height was brought to our trauma center. He sustained a radius shaft fracture combined with divergent elbow dislocation and distal radioulnar joint dislocation (Fig. 3a–d). Under general anesthesia, the humeroulnar joint was reduced by traction. Internal fixation of radius shaft was treated with the locking plates fixation. Furthermore, the distal radioulnar joint was reattached with an endobutton fixation (Fig. 3e–f). At the 7-month follow-up, the patient received an examination of the range of motion and showed a good clinical outcome (Fig. 3g–j).

A 35-year-old female presented pain in her hand and elbow with diffuse tenderness and swelling due to a fall from a 4 m height. On conventional X-rays radiograph, distal radius and radius head were comminuted. Additionally, posterior dislocation of the proximal ulna with displaced radius head fracture was a sign of convergent elbow dislocation (Fig. 4a–f). Initial closed reduction under general anesthesia for elbow dislocation was unsuccessful, and open reduction was performed to unlock the radius head, which was stuck with soft tissue. ORIF of distal radius fracture was received after replacement of radius head and repair of lateral collateral ligament. The patient was immobilized using an above-the-elbow plaster for 6 weeks (Fig. 4d–g). There were no symptoms of neurologic dysfunction at the follow-up. At the last follow-up, the patient had a whole range of motion in her elbow and wrist (Fig. 4k–p).

## Discussions

In 1952, Odnea et al. first reported the Monteggia and Galeazzi fractures at one side. They found the fracture mechanism: Two forces in opposite directions affected the same forearm, which was extremely rare [3, 6, 33–35]. In 1994, Jupiter et al. summarized 10 patients with complex bipolar fracture–dislocation of the forearm (“floating radius”) and observed radial instability as a standard feature [7].

Early diagnosis of the forearm subluxation/dislocation determined the subsequent surgical procedure. Preoperative radiographs were essential, including standard anteroposterior and lateral examination of the wrist and elbow joints to avoid the missed diagnosis of radioulnar joint dislocation. For example, anteroposterior radiographs of the elbow showed mismatching between the humeroulnar and humeroradial joints. The lateral radiographs showed that the radial head deviated from the center of the humeral head, suggesting subluxation of the radial head. Anteroposterior radiographs of the wrist showed the separation of the distal radioulnar joint over 2 mm, and the lateral radiographs showed the ulnar beyond the distal radius by more than 1/2, indicating distal radioulnar joint dislocation.

In addition, MRI was necessary to examine soft tissue injury. Tear of the interosseous membrane was a significant challenge for the stability of forearm rotation, ensuring that the hand was sited in a functional position for daily life, including eating, using tools and writing. In a report by Razak et al., the dynamic displacement of the interosseous membrane was found tiny by comparing the torque variation between pronation and supination of the interosseous membrane in a cadaver, suggesting that the interosseous membrane provided static longitudinal stability [36]. The attachment site of the central interosseous membrane demonstrated that it played an essential role in forearm longitudinal and rotational stability.

By far, reconstruction of the forearm interosseous membrane was a controversial topic. Some authors suggested enhancing the stability of the proximal radioulnar joint [37]. However, we considered that the tremendous clinical outcome could be achieved with a complete repair of the proximal radioulnar ligament and early rehabilitation. The surgeon could not observe the condition of the interosseous membrane until MRI evidence was confirmed, which was not a routine examination. In addition, excessive soft tissue dissection resulted in additional iatrogenic injury when internal fixation was used in shaft fractures. Latent damage of the interosseous membrane brought out postoperative dislocation/subluxation of the radioulnar joint. Therefore, it was emphasized that the soft tissue between the ulna and radius should be preserved and that joint matching should be thoroughly tested during the operation.

In adults, convergent and divergent elbow dislocations were a series of rare injuries [36]. Convergent elbow dislocation always occurred when the elbow was slightly bent and the hand was fully extended. The axial force resulted in an enormous valgus strain. The violence led to the ulnar collateral ligament tear and shear forces on the radial head, which generated a fracture before the radius rotated to the medial side of the forearm. The divergent dislocation was divided into an anteroposterior type and a mediolateral type. In the former type, the ulna coronoid was removed posteriorly to the olecranon fossa and the radial head was removed anteriorly to the coronoid fossa simultaneously. In contrast, the distal humerus was embedded between a medially displaced ulna and a laterally displaced radius head. Our treatment for these two types of dislocation focused on complete ligament repair and adequate external fixation.

Criss-Cross and Essex-Lopresti trauma were widely accepted as bipolar forearm injuries. However, our data excluded these conditions. Criss-Cross trauma was a dislocation (or injury) of the proximal and distal radioulnar joints that occurred when the radius was displaced along with central interosseous membranes. Previous articles considered Criss-Cross trauma as a dislocation of the radioulnar joint without a fracture [12]. Essex-Lopresti trauma included a fracture of the radial head, dislocation of distal radioulnar joint and tear of the central interosseous membrane. The radius instability was attributed to longitudinal displacement, resulting in severe elbow, wrist and forearm dysfunction. We considered Essex-Lopresti trauma as a specific type of bipolar fracture–dislocation of the forearm, requiring an entire tear of the central interosseous membrane and displacement of the radius. However, the patients in our study did not have evidence that the central interosseous membrane was destroyed.

There were many reports on patients with dislocation of the elbow and wrist joint, which was regarded as a type of bipolar dislocation of the forearm. However, because of simultaneous dislocation of the proximal and distal radioulnar joint, we could not figure out the stability of the forearm. Therefore, in this study, we also excluded “floating forearm.”

Our study proposed the definition of bipolar fracture–dislocation of the forearm: A trauma occurs when both proximal and distal radioulnar joints are dislocated with at least one radius and ulna fracture.

It was well known that open trauma and dislocation of the forearm should be treated emergently. However, there was no clear consensus available. The surgical approach depended on the fracture location and the surgeons' preference. Bipolar fracture–dislocation of the forearm was individualized due to diverse damage characteristics and morphologies. However, some principles could be proposed.

The treatment guideline for fracture–dislocation trauma was a complete dislocation reduction, which was unnecessary for long-term immobilization (less than 3 weeks for elbow, less than 4 weeks for wrist, and less than 6 weeks for ulnar and radial external fixation). Most enrolled patients in this study followed the above principles and achieved good clinical function. The fracture was treated based on joint stability, providing the best position to achieve an ideal fracture alignment after joint reduction. On the contrary, incomplete reduction often led to poor clinical outcomes. Prolonged postoperative Kirschner wires or plaster fixation to avoid re-dislocation of the joint was considered a common cause of subsequent elbow stiffness and limited mobility.

The treatment principles were as follows: (i) The anterior dislocation of the radial head in Monteggia Bado I fracture was not stable, which required increasing the backward radian slightly. Rarely, because soft tissue insertion often occurred in convergent elbow dislocation, open reduction was performed to repair the annular ligament. (ii) The posterior dislocation of the radial head was treated after anatomic reduction in the ulna fracture. The function of posterolateral rotation was achieved in repairing the lateral collateral ligament by fixing it with suture anchors or perosseous drilling. (iii) After anatomic fixation for distal or shaft radius fracture, the radioulnar joint was usually stable, which allowed exercise initiating at 3 days postoperatively. Otherwise, Kirschner wires external fixation of the distal radioulnar joint in supination position was required for 4–6 weeks. (iv) The stability of the humeroulnar joint was fixed with closed reduction, which should be double-checked according to the elbow extension test. Conversely, when conditions were unfavorable, hinged external fixation of the elbow was performed. (v) Excessive dissection of the soft tissue from the ulna and radius should be avoided. It was necessary for maintaining length and anatomic matching between radius and ulna, which was beneficial to interosseous membrane healing.

There were some limitations in our study. First, different surgeons were involved in our retrospective study in the last 10 years. Surgical techniques were not standardized, which might influence our results. However, the concept of priority in dealing with dislocated joints and early rehabilitation has been reserved in our institutions. Second, some patients were lost by more than 1-year follow-up. Therefore, longer follow-up likely figures out more complications and reoperations in these patients.

## Conclusions

Bipolar fracture–dislocation of the forearm is a complex injury and its treatment principles have not reached a consensus. Thus, comprehensive analysis and careful consideration are required before surgeons perform the surgery. The primary objective for bipolar fracture–dislocation of the forearm is to maintain dislocated/subluxation joint stability. It is necessary to ensure linear and positional alignment to obtain proper soft tissue tension and joint matching. Intraoperative fracture fixation depends on stable reduction for dislocation. Patients with dislocation reduction and early rehabilitation always present good function.

## Data Availability

The datasets used and/or analyzed during the current study are available from the corresponding author on reasonable request.
